# Endogenous complement human serum bactericidal assay (enc-hSBA) for vaccine effectiveness assessments against meningococcal serogroup B

**DOI:** 10.1038/s41541-021-00286-8

**Published:** 2021-02-23

**Authors:** Annett Kleinschmidt, Kumaran Vadivelu, Laura Serino, Nina Neidig, Bertrand de Wergifosse

**Affiliations:** 1 GSK, Marburg, Germany; 2 GSK, Siena, Italy; 3 GSK, Rixensart, Belgium

**Keywords:** Immunology, Vaccines, Meningitis

## Abstract

Immunogenicity of vaccines against meningococcal serogroup B (MenB) has been assessed pre-licensure with a human serum bactericidal activity assay (hSBA), tested against small numbers of strains. We report the qualification/validation of an alternative qualitative hSBA which uses endogenous complement (enc-hSBA) present in the vaccinee’s serum. Serum samples were collected from adults pre-vaccination and post-vaccination with the 4-component MenB vaccine (4CMenB). A representative panel of invasive isolates and 4 antigen-specific indicator strains were used in qualification experiments. Each strain was tested in ≥3 experiments with pre/post-vaccination sera to evaluate intermediate precision. A 110-strain panel and the 4 indicator strains met qualification criteria, demonstrating assay precision. Assay robustness, specificity and sensitivity were demonstrated using the 4 indicator strains. Enc-hSBA is highly standardized, allows testing across large panels of epidemiologically-relevant MenB strains, and accounts for complement activity differences between vaccinees. Therefore, enc-hSBA enables a more accurate estimation of effectiveness for vaccines against MenB.

## Introduction

*Neisseria meningitidis*, a human pathogen, is a prominent cause of bacterial meningitis and sepsis worldwide. Even with appropriate medical treatment, invasive meningococcal disease (IMD) has a case fatality rate of 4–20%^1^. In addition, up to 20% of survivors experience long-term debilitating sequelae, such as hearing loss, neurological impairment and amputations^2^. IMD primarily affects infants, but secondary peaks in incidence are also observed in adolescents and older adults^2,3^. The consequences of IMD may be significant not only for those directly affected, but also their families and the healthcare system^4^. Prevention through vaccination is the best defense against this aggressive disease that leaves little time for intervention.

The epidemiology of IMD is dynamic, as the prevalence of meningococcal serogroups changes over time and new strains emerge frequently. Moreover, serogroup prevalence can vary substantially from one region to another^5^. Of the 12 *N. meningitidis* serogroups identified to date, 6 are responsible for almost all instances of IMD: meningococcal serogroup (Men) A, MenB, MenC, MenW, MenY, and MenX^2^. In many industrialized countries, MenB is currently the predominant cause of IMD^6,7^.

Vaccines currently available against MenA, MenC, MenY, and MenW-caused IMD contain capsular polysaccharides, either alone or—more often—conjugated to a carrier protein. By contrast, the MenB capsular polysaccharide displays poor immunogenicity due to its similarity with human glycoproteins^8^. Therefore, MenB vaccines containing subcapsular proteins were developed^9^. Two protein-based vaccines are now licensed for use in several countries worldwide: a 4-component MenB vaccine (4CMenB; *Bexsero*, GSK) and a bivalent vaccine (rLP2086, *Trumenba*, Pfizer). 4CMenB contains 4 antigenic components: factor H binding protein (fHbp) variant 1, the Neisserial Heparin Binding Antigen (NHBA), *Neisseria* adhesin A (NadA), and outer membrane vesicles (OMV) expressing porin A (PorA) serosubtype P1.4^10^. rLP2086 includes 2 lipidated proteins, belonging to fHbp variants 1 and 3^11^.

Due to the low incidence of IMD, it is not feasible to conduct studies that are sufficiently powered to demonstrate the efficacy of meningococcal vaccines, as this would require an impractical number of participants. Instead, for licensure purposes, the immunogenicity of 4CMenB and rLP2086 was demonstrated in clinical trials in terms of percentages of participants with serum bactericidal activity (SBA) above a specific threshold following vaccination. Immunogenicity was tested with an SBA assay using exogenous human complement from seronegative donors (hSBA)^12,13^. An hSBA titer of at least 1:4 is widely accepted as a surrogate marker of protection against meningococcal disease^14^. At present, the immunogenicity of MenB vaccines is measured against a limited number of strains. However, the susceptibility of a MenB strain to vaccine-induced antibodies depends on the antigenic similarity of the bacterial and vaccine antigens, as well as the amount of antigen expressed on the surface of the invading meningococci. Antigen sequence and level of expression are known to vary across MenB strains, over time and from one geographical region to another^15,16^. Therefore, since it is not possible to test all circulating strains, the effectiveness of the MenB vaccines can be estimated by assessing the protection afforded against a panel of MenB strains as large and as relevant as practicably possible. We developed a qualitative hSBA-based assay that uses endogenous complement present in the serum of each individual (enc-hSBA). This enables testing on broad panels of epidemiologically representative MenB strains to provide a more accurate assessment of vaccine effectiveness. Here, we present the qualification and validation of enc-hSBA, using a large number of MenB strains. Box 1 displays a plain language summary of this article for the reader.

## Results

**Fig. 1:**
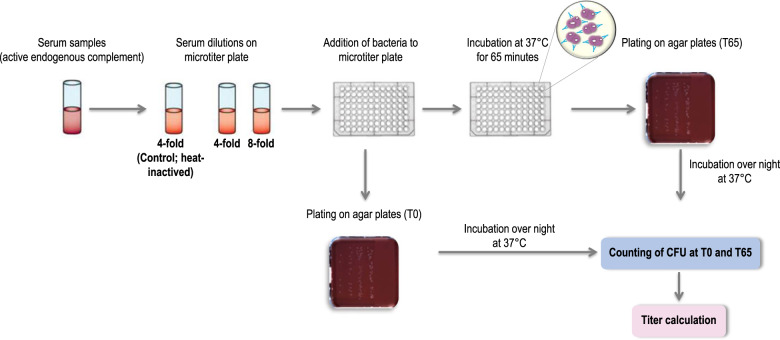
Enc-hSBA: assay principle. Audioslides on the enc-hSBA assay principle are available on the Figshare repository (10.6084/m9.figshare.13547006). Enc-hSBA, serum bactericidal activity assay using endogenous human complement; T65, after 65 min of incubation; T0, time 0; CFU, colony-forming units.

Enc-hSBA measures vaccine-induced antibody mediated killing (≤50% colony forming units [CFU] at time [T] 65 (after 65 min of incubation) compared to T0), at pre-defined dilutions (4-fold and 8-fold) of the serum samples (Fig. 1). The selection of these dilutions was done based on the observation that the concentration of human complement proteins may not be sufficient to induce bacterial killing at dilutions between >8-fold and 16-fold. The enc-hSBA titer is established based on a double determination strategy. The outcomes of experiments at both 4-fold and 8-fold dilution are taken into account, and samples are reported with titers of 1:4, 1:8, or <1:4 (no killing) (Table 1).

**Table 1:** Possible outcomes of enc-hSBA measurements in an individual test with 4-fold and 8-fold diluted samples used for titer determination.

	CFU count at T65	
Reported titer	4-fold dilution	8-fold dilution
<1:4	>50% of T0	>50% of T0
1:4	<50% of T0	>50% of T0
1:8	<50% of T0	<50% of T0
Prozone	>50% of T0	<50% of T0

Enc-hSBA, serum bactericidal activity assay using endogenous human complement; CFU, colony forming units; T65, after 65 min of incubation; T0, time 0.

## Bacterial strain selection

A large panel of strains collected from the United States (US) between 2000 and 2008 were tested for qualification with enc-hSBA, as described below. The strains were randomly selected from a total of 442 MenB isolates^17,18^ obtained from the Centers for Disease Control and Prevention (CDC) repository of invasive strains, which were systematically collected in the US through the National Notifiable Diseases Surveillance System Active Bacterial Core surveillance (www.cdc.gov/ncidod/DBMD/abcs/). Of the 442 strains, 140 were randomly selected and 139 were supplied to the laboratory.

Four strains which enable measuring of SBA directed mainly against one of the 4 major vaccine antigens were also used in qualification and validation experiments. These indicator strains were used for immunogenicity measurements and are specific to each major component of 4CMenB: M14459 for fHbp, M07-0241084 for NHBA, 96217 for NadA, and NZ98/254 for PorA. Their characteristics are provided in Table 2.

**Table 2:** Characteristics of the 4 indicator strains.

Strain	Target antigen	Country, year of isolation	Phenotype	CC (ST)	4CMenB antigens		
					fHbp	NHBA	NadA
M14459^1^	fHbp	United States, 2005	B:NT:P1.22,9	NA (2048)	**1.110**	19	–
M07-0241084	NHBA	United Kingdom, 2007	B:4:P1.19,15	41/41 (1097)	2.302	**31**	–
96217	NadA	Canada, 1996	B:2b:P1.5,10	8 (153)	2.16	20	**2/3**
NZ98/254	PorA	New Zealand, 1998	B:4:P1.7-2,4	41/44 (42)	1.14	2	–

The primary target antigen of bactericidal killing, CC and ST are provided for each supplemental test strain. The PorA serosubtype, fHbp subvariant, NadA allele/peptide, and NHBA peptide are given for each strain. The fHbp nomenclature is as used in the *Neisseria* PubMLST database (http://pubmlst.org/neisseria/). 4CMenB antigen presence or anticipated cross-reactivity is presented in bold.

*CC,* clonal complex; *4CMenB,* 4-component meningococcal serogroup B vaccine; *NA,* not assigned; *ST,* sequence type; *fHbp,* factor H binding protein; *NadA*, *Neisseria* adhesin A; *NHBA,* Neisserial Heparin Binding Antigen; *PorA,* porin A.

^1^The indicator M14459 was also part of the 442 strains from which the 110-strain panel was randomly selected, but was not included in the selection.

## Qualification of the 110-strain panel

In intermediate precision experiments, each strain was tested in triplicate, using the same set of serum samples, by at least 2 different operators on at least 2 different days. For each strain, each of the 3 experiments was conducted using the same panel of 20 serum samples, collected pre-vaccination (10) or post-vaccination (10) with 4CMenB. The objective of the qualification process was to demonstrate consistency of results in the triplicate testing. Consistency was demonstrated if the same result was observed for ≥75% of serum samples (15 of 20 tested samples for each strain).

**Fig. 2:**
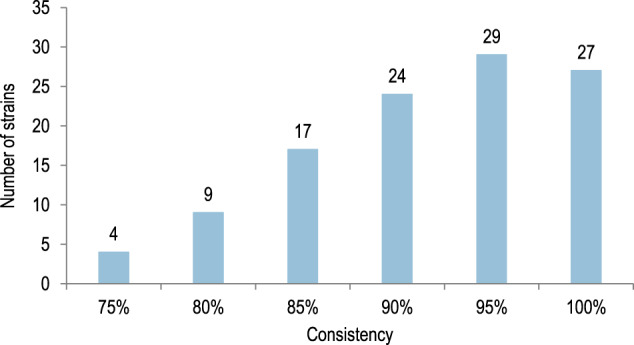
Qualification of the 110-strain panel in enc-hSBA: distribution of the MenB strains by consistency (percentage of samples with the same result obtained in triplicate testing). Enc-hSBA, serum bactericidal activity assay using endogenous human complement; MenB, serogroup B meningococcus.

A total of 139 strains were investigated. Prior the qualification process, all strains were tested for their ability to grow under laboratory conditions; strains with growth limitations were excluded from hSBA testing. Of the 139 strains, 13 were excluded from qualification due to growth limitations: inadequate growth in the liquid Müller-Hinton medium (5 strains) or clumping during bacterial growth (8 strains). Of the 126 remaining strains, 114 were tested sequentially in enc-hSBA in order to reach the pre-set goal of 110 qualified strains. Four of the 114 strains tested in enc-hSBA did not meet consistency criteria. For most of the strains meeting the qualification criterium (106/110), consistency was demonstrated for ≥80% of serum samples tested (Fig. 2).

The percentages of samples with bactericidal activity for individual strains at 4-fold dilution varied from 0% to 100% pre-vaccination and from 6.7% to 100% post-vaccination. The average percentage of samples with bactericidal activity was 42.3% at pre-vaccination and 82.8% post-vaccination, across all 110 qualified strains.

The percentages of samples with bactericidal activity for individual strains at 8-fold dilution varied from 0 to 100%, regardless of timepoint of collection. The average percentages of pre-vaccination and post-vaccination samples with bactericidal activity were 22.5% and 65.9%.

## Assay validation using the 4 MenB indicator strains

### Intermediate precision

Intermediate precision was assessed to demonstrate consistency, according to the same criteria used for the 110-strain panel qualification. For each of the 4 indicator strains, 20 pre-vaccination and 20 post-vaccination human sera were used resulting in 40 samples tested in triplicate. All 4 indicator strains met the pre-established criteria to show intermediate precision, with 90% (M14459), 85% (M07-0241084), 93% (96217), and 83% (NZ98/254) consistency across serum samples tested. A more detailed statistical assessment of intermediate precision based on the % CFU counts is described in the Supplementary Note 1.

### Limit of blank

The limit of blank (LOB) was defined as the lowest % CFU count observed when repeatedly measuring a blank sample (with the blank giving full bacterial count with 100% CFU). Conversely, any % CFU count <LOB contains with high probability immunologically active antibodies.

The CFU counts of the heat-inactivated controls were used to represent blank (antibody negative) samples. Heat-inactivated complement controls for all measurements (20 samples per strain × 3 tests × 2 replicates) of the intermediate precision, performed in qualification experiments (Supplementary Note 1), were evaluated.

For each strain, the LOB was calculated as one-sided prediction limit with respect to two different type I error rates, for α = 0.95 and for α = 0.99, across all bacterial counts observed for the heat-inactivated samples at 4-fold dilution. The one-sided lower prediction limits (LPL) were calculated from Eq. (1):

1 $${\mathrm{LPL}} = Yn - t_{1 - \alpha ,n - 1}s_n\sqrt {1 + \frac{1}{n}}$$

where *Y*_*n*_ and *s*_*n*_ are arithmetic mean and empirical standard deviation calculated from the existing *n* data points, and *t*_*1-α,n-1*_ is the (1–*α*)-quantile of the *t*-distribution with *n*-1 degrees of freedom. All calculations were performed for square root-transformed data and then reconverted to the original units.

For all strains, the 99% LPL was above 50% (Table 3), indicating that a sample for which a value <50% CFU is observed during routine testing is with very high probability not a blank. Since an enc-hSBA titer of 1:4 is only assigned if both determinations in the duplicate measurement are positive, the chance for a false positive result was reduced further to approximately 0.01%. Therefore, the assay was considered as suitable for a certain strain if the corresponding 99% LPL lies above the cut-off of 50%.

**Table 3:** Lower one-sided 99% prediction limits of survival rates for each indicator strain.

Strain	*N*		Mean over sqrt (% CFU)	SD over sqrt (% CFU)	Average (% CFU)	LPL (%)
M14459	120		9.743	0.6764	95	66
M07-0241084	120		10.470	1.2793	110	55
96217	120		10.436	0.8737	109	70
NZ98/254	120		10.318	1.1196	106	59

Note: All calculations were performed for square root-transformed data and then reconverted to the original units (% CFU).

*N,* number of measurements; *sqrt,* square-root; *CFU,* colony-forming units; *SD,* standard deviation; *LPL,* one-sided lower prediction limit.

### Robustness

Robustness experiments were performed using 2 indicator strains (M14459 and NZ98/254, specific for the fHbp and PorA components of 4CMenB).

A potential effect of the sample preparation/storage on the stability of complement activity in serum samples was investigated, as the current process involves short-term storage of the samples at −20 °C. The samples (handled as described in the Sample management subsection) were tested in enc-hSBA after being stored several times at −20 °C, each time for maximum 3 h. Storage of samples up to 6 times at −20 °C was found to have no significant impact on the intrinsic complement activity of serum. In addition, up to five freeze-thaw cycles (1 h at room temperature followed by re-freeze at −80 °C for at least 2 h) were shown to have no influence on complement activity and consequently, on the enc-hSBA titer (data not shown).

To evaluate bacterial growth within accepted assay range, 10 serum samples were selected and tested in enc-hSBA, using bacteria harvested at the lower (optical density at 620 nm [OD_620nm_] = 0.650) or upper (OD_620nm_ = 0.900) end of the accepted OD_620nm_ range for bacterial growth in enc-hSBA. In this experiment, serum dilutions from 2-fold to 16-fold were used and the corresponding titers were calculated as for the traditional hSBA (with exogeneous complement)^14^, using extrapolation. No significant difference between titers calculated at OD_620nm_ = 0.650 or OD_620nm_ = 0.900 was observed (data not shown).

### Specificity

Specificity was evaluated by inhibition experiments, which aimed to demonstrate that killing of the 4 indicator strains was induced by vaccine-specific antigens. The inhibition experiments were performed by adding 4CMenB proteins to the tested serum sample. As the 4CMenB proteins can bind vaccine-induced antibodies, this would lead to a lower antibody availability for binding the bacterial strain’s proteins on the bacteria surface, and consequently, a decrease in the enc-hSBA titer indicating reduction of bactericidal killing.

For each strain, 2 post-vaccination serum samples with a positive MenB titer (≥1:8) were selected. A pool of recombinant proteins with OMV was added to the undiluted sample and the aliquot was incubated overnight (16–24 h) at +4 °C and then measured in enc-hSBA. In the added protein pool, 4CMenB components were mixed in a fHbp: NHBA: NadA: OMV ratio of 1:1:1:2, leading to final concentrations of 111.1 μg/mL for fHbp, NHBA, and NadA and 166.7 μg/mL for OMV.

**Fig. 3:**
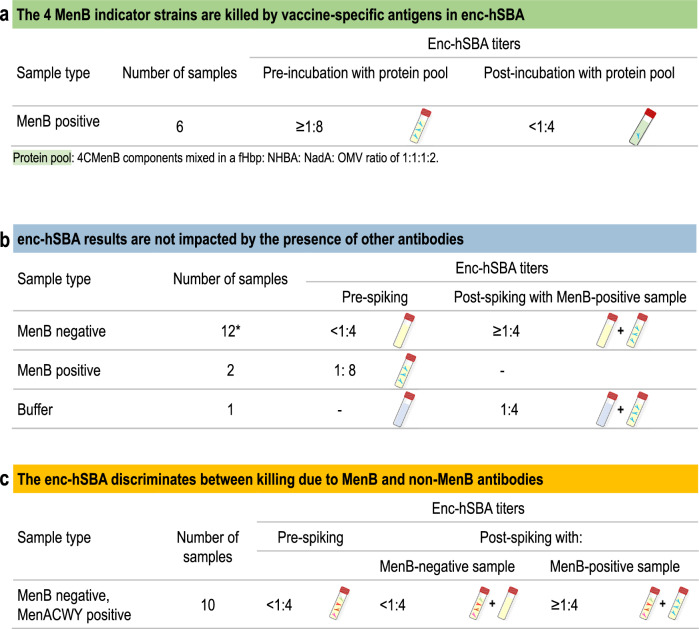
Enc-hSBA specificity. Enc-hSBA specificity evaluated by inhibition experiments (**a**) andsensitivity evaluated by spiking experiments (**b**, **c**), using testing against the 4 indicator strains. Enc-hSBA, serum bactericidal activity assay using endogenous human complement; MenB, serogroup B meningococcus; 4CMenB, 4-component meningococcal serogroup B vaccine; fHbp, factor H binding protein; NadA, *Neisseria* adhesin A; NHBA, Neisserial Heparin Binding Antigen; OMV, outer membrane vesicles; MenACWY, serogroup A, C, W, and Y meningococci. Note: *From 2 different sets of experiments.

Adding 4CMenB proteins to positive MenB sera (titer ≥ 1:8) led for all 4 test strains to a titer reduction to <1:4, demonstrating vaccine-specific killing (Fig. 3a).

### Sensitivity

Sensitivity was evaluated by spiking experiments, designed to demonstrate whether the enc-hSBA results are affected by the presence of other antibodies and to show that the assay can discriminate between killing due to MenB and non-MenB antibodies (Fig. 3b, c).

In a first set of experiments, 2 MenB negative (titer < 1:4) serum samples and 1 buffer sample were selected and spiked with 2 samples that contained MenB vaccine specific antibodies (positive serum). As a control the 2 negative samples and the MenB positive samples were tested alone. For each tested strain, the 2 negative samples showed a titer of <1:4 and the 2 MenB positive samples showed a titer of 1:8. Next, the negative sera and the buffer were each incubated in a 1:1 ratio with the MenB positive sample. The measured enc-hSBA titer for the spiked buffer samples for all 4 strains was 1:4, while the titer of the negative sera spiked with the MenB positive samples was ≥1:4 for all 4 indicator strains. Therefore, the bactericidal activity of the MenB-positive samples was retained in the presence of negative sera.

In a second set of experiments, 10 MenB negative samples were spiked with 1 MenB positive sample. For all MenB negative samples without spiking, a titer of <1:4 was observed while the titers increased to ≥1:4 after spiking, for all 4 indicator strains. Therefore, the titer of negative samples was increased in the presence of positive sera.

In a third set of experiments, 10 serum samples with negative MenB titer but a positive MenACWY titer (≥1:8, for at least one serogroup) were investigated using the 4 indicator strains. The MenACWY positive samples were spiked (1:1) with a MenB negative sample (titer < 1:4) and separately with a MenB positive sample (titer ≥ 1:8). In enc-hSBA, MenACWY positive samples spiked with a MenB negative sample showed a titer of <1:4, while spiking MenACWY positive samples with a MenB positive sample shows a titer of ≥1:4 for all 4 indicator strains. Therefore, the presence of the MenACWY capsular polysaccharide antibodies does not affect the enc-hSBA measured complement-mediated killing by MenB-specific antibodies.

## Discussion

This paper presents the development of a qualitative method which can be applied to measure the effectiveness of MenB vaccines through the evaluation of complement-mediated bactericidal killing against a large panel of MenB strains, using the vaccinated individuals’ sera as a source of complement. The qualification of enc-hSBA for a large panel of 110 invasive MenB strains was performed. The method was then further validated using 4 indicator strains specific to each 4CMenB antigen.

The qualified 110-strain panel was randomly selected from 442 invasive MenB strains collected by the CDC throughout 10 states in the US over a period of 8 years. The 442-strain panel was weighted down with respect to higher representation of fHbp subvariant 1.1 due to a MenB− and overall meningococcal disease outbreak occurring in Oregon in this period^17^. Invasive meningococcal strains collected in the same timeframe (2000–2008) were previously genetically characterized and evaluated in terms of prevalence and genetic diversity of the fHbp, NHBA, and NadA antigens^18^. In addition, comparison with a panel of strains collected during 1989–1999 did not indicate any substantial changes in the distribution or composition of fHbp and NHBA subvariants or the proportion of isolates with NadA presence^18^. The panel of 442 MenB strains was also used to estimate 4CMenB vaccine coverage in the US with the Meningococcal Antigen Typing System (MATS), predicting that 91% (95% confidence interval [CI]: 72–96) of the circulating strains would be covered by 4CMenB^17^. Using the genetic MATS (gMATS), which combines antigen genotyping with MATS assessment, 85.4% strain coverage (lower limit–upper limit: 76–95) was predicted for 4CMenB across the same panel^19^.

Since the 110-strain panel used for qualification with enc-hSBA was randomly selected from the 442 MenB strains, it was considered to be adequately representative of the larger panel, also in terms of the most frequent 4CMenB genotypes likely to play a major role in the outcome of killing in enc-hSBA. Randomization also ensured minimization of potential bias derived from other factors critical for killing of MenB strains in enc-hSBA, either known (e.g., clonal complex) or unknown. Furthermore, the 110-strain panel was compared with strains collected during 2015–2017, following the implementation of the Enhanced Meningococcal Disease Surveillance (EMDS) by the CDC. The distribution of clonal complexes and MenB vaccines antigen genotypes were found to be comparable between the 2 panels^20^, indicating that the 110-strain panel remains representative of invasive MenB strains circulating in the US in terms of factors which may impact assessment of SBA. Therefore, the qualification of the 110-strain panel for enc-hSBA provides the means to more accurately assess protection afforded by vaccines containing MenB components, with estimations which continue to be relevant to the current epidemiology of MenB-caused IMD in the US.

When validated with the 4 MenB indicator strains, enc-hSBA showed good precision, with a pronounced transition from negative to positive of samples. Furthermore, the established LOB above 50% bacterial count ensures that the probability of false positive samples is limited to ~0.01%. Assay specificity was also shown using the 4 indicator strains, which were killed by 4CMenB specific antibodies in the enc-hSBA. Moreover, the presence of antibodies not related to MenB did not inhibit the killing in enc-hSBA.

With the successful qualification of the method for a panel of 110 strains and its validation, enc-hSBA is to date the most suitable bactericidal assay to estimate the effectiveness of vaccines containing MenB components. The panel has already been used to generate clinical data: in a trial evaluating vaccination of adolescents with 2 and 3 doses of an investigational MenABCWY vaccine containing 4CMenB components, vaccine effectiveness was determined as the proportion of MenB strains killed in enc-hSBA at 4-fold dilution by samples collected from MenABCWY-immunized compared to MenACWY-immunized individuals. Across the panel of 110 US MenB strains, vaccine effectiveness was 67% (95% CI: 65–69) after 2 doses and 71% (95% CI: 69–73) after 3 doses of MenABCWY vaccine^21^. Enc-hSBA is to date the most exhaustive method to assess vaccine effectiveness and can be complemented by other tools to assess strain coverage predictions, such as MATS or genetic MATS^19^ for 4CMenB, or the flow cytometric meningococcal antigen surface expression (MEASURE) assay for rLP2086^22^. For instance, genetic MATS can be applied to evaluate coverage of strains based on antigen genotyping data only, without requiring a live isolate^19^. However, as it is based on MATS, which is known to underestimate SBA killing^23,24^, gMATS potentially provides a conservative measure of 4CMenB strain coverage. The use of enc-hSBA can be expanded beyond the generation of clinical data, to the monitoring of vaccines’ performance during or after an outbreak event, or to anticipate their impact on the burden of IMD over time, as MenB strains continue to evolve. So far, real-world evidence is only available for 4CMenB, the only vaccine to be used as part of a national immunization program, in the United Kingdom. Since 2015, over 3 years from vaccine introduction, the incidence of MenB-caused IMD was reduced by 75% in the cohorts fully eligible for vaccination and vaccine effectiveness among children receiving the full 3-dose schedule was estimated to be 59.1% (95% CI:−31.1– 87.2). When assuming that 4CMenB provides protection only against MATS-positive strains, an adjusted vaccine effectiveness of 71.2% was estimated against 4CMenB-preventable MenB disease^25^. 4CMenB has also been used in a large vaccination campaign, following an outbreak occurring in the Saguenay-Lac-Saint-Jean region of Quebec, Canada. Over a period of 4 years from campaign launch, the incidence of MenB-caused IMD in the population targeted for vaccination (individuals ≤20 years of age) declined significantly and a 86% (95% CI: −2– 98) decrease in MenB-IMD risk was estimated in the region^26^.

However, enc-hSBA has several potential limitations. The method is a qualitative assay, and results can only be reported as one of 3 possible outcomes (titer of 1:4, 1:8, and negative). Therefore, in contrast to the classical hSBA, the true titer for each sample tested with enc-hSBA is not known, which precludes the statistical evaluation of geometrical mean titers or variations over time in titers above the assay’s threshold for positivity. A large number of tests have to be performed to cover all strains in the qualified panel, which translates into a high operational burden. Moreover, because a limited volume of serum is available from each individual, not all strains can be tested with the same sample; however, this is not likely to impact the generalizability of the results, since strains are randomly assigned to each serum sample before testing. In addition, as enc-hSBA is based on the use of the individual’s own complement, bactericidal activity might be impaired for patients with asplenia, complement deficiencies, and/or persons receiving treatments that impact the complement system.

Notwithstanding these limitations, enc-hSBA is a highly-standardized method, allowing reproducible measuring of bactericidal killing against a large variety of circulating MenB strains. The method can also be applied to new strains, pending their qualification, thus allowing testing vaccine effectiveness across large panels of epidemiologically relevant strains, selected as representative of the continuously-evolving MenB strains. More importantly, enc-hSBA is not vaccine-specific and can be applied to other MenB-containing vaccines after qualification, which would allow a direct comparison between vaccine effectiveness estimates for different vaccines.

The use of the endogenous human complement in enc-hSBA bypasses the need for a suitable and sufficient exogenous complement source. To be used in hSBA testing of immunogenicity against meningococcal strains, human complement should be free of antibodies capable to contribute to bacterial killing, while still maintaining normal complement activity. The complement should also be sourced from individuals with no prior immunity (acquired naturally or otherwise) to meningococcus. In addition, its standardization can prove challenging^27^. In enc-hSBA, the laborious and time-consuming step of identifying suitable human complement sources is not necessary. The use of endogenous human complement also means that the assay provides a direct measure of individual bactericidal killing of MenB strains, as SBA can differ from one individual to another, due to differences in the vaccine-induced immune response, as well as in individual complement composition/activity^28^. Enc-hSBA accounts for this source of variability and therefore its added value is its use for the assessment of a responder-based vaccine effectiveness. In addition, enc-hSBA could also be used to test bactericidal killing induced by MenACWY vaccination against non-MenB strains, although this has not yet been investigated.

In conclusion, enc-hSBA is a robust qualitative method that allows testing against large panels of circulating MenB strains and provides epidemiologically-relevant predictions of strain coverage and the respective vaccine effectiveness across MenB strains. Enc-hSBA could be used to evaluate the performance of vaccines against MenB-caused IMD.

## Methods

### Bacterial strains

For all strains, master seeds and working seeds were prepared by growing the bacteria overnight on solid chocolate agar and subsequently preparing aliquots for storage at −80 °C. For preparation of the working seed, a master seed was sub-cultured and cultivated overnight on chocolate agar followed by preparation of aliquots in glycerol-media. Working seeds and master seeds were stored at −80 °C. For each experiment, a new working seed aliquot was thawed and cultured.

### Assay conditions

Enc-hSBA, based on the hSBA described by Goldschneider et al.^14^, uses endogenous complement. Individual colonies of MenB bacteria grown on chocolate agar overnight are picked and suspended in liquid Müller-Hinton and grown in the logarithmic phase (OD_620nm_ between 0.65 and 0.9) for 90–180 min. Each serum sample is tested in duplicate at 4-fold and 8-fold dilution. The serum samples are not heat-inactivated, to maintain intrinsic complement activity. As control, each individual serum sample is also tested following heat-inactivation. After preparation of the microtiter plate with diluted sera, bacteria from a fresh log-phase liquid culture are added. The microtiter plate is incubated for 65 min at 37 °C. Aliquots of each well (test reactions and controls) are transferred onto chocolate agar and incubated overnight. Counting of CFU is performed with an automated Colony Counter System. For each sample, the mean number of CFUs at T65 is compared with the number of colonies at test start (T0) (Fig. 1).

### Test controls

For the buffer control, the following acceptance criteria were defined: (i) the number of CFU at T0 must be 30–100 (to ensure a sufficient number of bacteria in each well), and (ii) the slope between the mean CFU calculated at T0 to T65 must be at least 0.9.

To ensure that any bactericidal activity observed is complement-mediated and that the serum sample does not have complement independent killing activity, each individual serum sample was heat-inactivated and tested at a 4-fold dilution. If the T65 CFU for the heat-inactivated sample was <75% of T0 CFU for both replicates or ≥75% of T0 for one replicate but <50% of T0 for the other, the sample was defined as toxic and tested again. If the same outcome was obtained, or additionally the T65 CFU was ≥75% of T0 for one replicate and ≥50%–<75% for the other replicate, the sample result was reported as toxic, with no valid result.

If the outcomes of the 2 determinations did not match (one positive and one negative), the result was considered inconclusive and the sample was retested. If, in the retest, the result was again inconclusive, a negative titer (<1:4) was reported. If the first and second determinations yielded titers of 1:4 and 1:8, respectively, then a titer of 1:4 was reported for the sample. A prozone effect was defined for samples which have higher bactericidal killing at the 8-fold (>50% of T0 CFU counts) than at the 4-fold (<50% of T0 CFU counts) dilution. All samples with unclear killing curves (at least one replicate with CFU counts lower at 8-fold than at 4-fold dilution) were re-tested. In addition, any samples with test abortion (due to human handling error, instrument failure, sample contamination, and unusual bacterial growth) were also retested.

In validation experiments using the 4 indicator strains, a positive (titer ≥ 1:4 or 1:8) and a negative (titer < 1:4) control were included for each assay and for each strain with predefined titers and acceptance criteria to monitor assay performance over time.

### Serum samples

Serum samples from healthy adults before and after vaccination were collected during completed clinical trials or from donors vaccinated with 4CMenB. Sera from the following sources were tested:

i. clinical trials NCT01478347 (study A) and NCT01911221 (study B), 2 phase 3b studies in which participants aged 18–65 years received 4CMenB according to a 0, 2-month schedule (pre-vaccination, and 1 month post-2nd vaccination samples);
ii. clinical trial NCT02305446 (study C), a phase 3b study in which adults 18–50 years of age received 4CMenB according to a 0,2-month schedule and agreed to donate their blood for use as a reference in SBA tests (pre-2nd vaccination and post-2nd vaccination samples);
iii. healthy adults 18–65 years old with no previous meningococcal vaccination history enrolled in a non-interventional study (study D) for collection of blood and plasma from selected SBA-negative donors for meningococcal assays (pre-vaccination samples);
iv. vaccinated individuals with a documented history of immunization of 3 doses of 4CMenB, donating blood at the Marburg (Germany) Siemens blood bank (post-vaccination samples);
v. clinical trial NCT02140762 (study E), a phase 2b study in which adolescents 10–18 years of age received MenABCWY according to a 0,2-month schedule^21^ (post-vaccination samples).

The origin of serum samples used for qualification/validation experiments is presented in Supplementary Table 1. An informed consent form which included permission for potential (re-)use of the biological samples was obtained for participants in all the clinical trials and the individual blood donors.

### Sample management

Sera collected from the clinical trials were separated in the day of blood sample collection and stored at −80 °C to preserve the intrinsic complement activity. Serum aliquots were sent on dry ice to the laboratory where enc-hSBA testing was performed. Prior to further aliquoting, 81-slot cryoboxes (one per each sample) were cooled on ice. All samples collected from the same individual were thawed at 37 °C for 5–10 min, pooled (if needed), and aliquoted (maximum 81 × 80 μL aliquots in 2 mL tubes placed in cryoboxes) on ice. After aliquoting, the aliquots and any left-over material were stored at −80 °C until the assay was performed. An interactive response technology (IRT) system was used to calculate the number of strains to be tested for each sample and to randomly assign sample aliquots to strains. Laboratory personnel sorted the samples for testing according to the blinded report generated with the IRT system and prepared sorting lists which document the strains to be tested, the location of the sample and available aliquots. Sorting and transfer of aliquots to test boxes for each strain was performed in a cold room at −20 °C.

Box 1 **What is the context?**

- *Neisseria meningitidis* serogroup B (MenB) accounts for a large proportion of invasive meningococcal disease cases.
- For vaccines against MenB, immune response pre-licensure is currently assessed by the human serum bactericidal assay (hSBA).
- The hSBA assay measures whether antibodies from people who have been vaccinated can kill MenB laboratory reference strains.
- hSBA requires seronegative exogenous complement from unvaccinated individuals and can therefore only be performed against a limited number of MenB reference strains.

**Manuscript highlights and take-home message**

- We describe an alternate method, called enc-hSBA, which is based on hSBA but uses the vaccinated person’s readily available sera as the complement source.
- Through validation and qualification, we show that enc-hSBA is a robust qualitative method that allows testing against a large panel of MenB strains.
- Therefore, enc-hSBA can measure immune responses against a wide variety of circulating MenB strains. It enables more accurate estimations of the protection afforded by MenB-containing vaccines.

## Supplementary information

Supplemental Information

## Data Availability

This manuscript does not disclose primary study data. The origin of serum samples used for the enc-hSBA qualification/validation experiments is presented in Supplementary Table 1. To request access to patient-level data for the primary studies and documents for this study, please submit an enquiry via www.clinicalstudydatarequest.com.
