# Supplementary material for: Endogenous complement human serum bactericidal assay (enc-hSBA) for vaccine effectiveness assessments against meningococcal serogroup B
Source: NPJ Vaccines. 2021 Feb 23;6:29. doi: 10.1038/s41541-021-00286-8 (PMC7902841; doi:10.1038/s41541-021-00286-8)
Supplement: Supplementary file 1 — Supplemental Information [file 41541_2021_286_MOESM1_ESM.pdf]

Supplementary Material

Note 1. Intermediate precision results

The variability of bacterial counts was evaluated across dilution ranges, in qualification experiments. All inconclusive and invalid results from the consistency experiments were removed such that exactly 12 individual bacterial counts (expressed as proportions of the CFU counts at T0) were available, for each sample (3 tests x 2 replicates x 2 dilutions). By analyzing the distribution of bacterial counts with dilution, by titer, it was observed that variances increase with bacterial count. In order to assess the impact of the variability on the range of inconclusive results around 50% bacterial survival, the average bacterial count across all 6 available results per sample was calculated as the best estimate of the “true” bacterial count, and the 6 individual results were represented versus the 6-fold average (**Supplementary Fig. 1**). According to this representation, samples with an average bacterial count around 20% or lower are consistently classified negative, while samples with a bacterial count around 80% or higher are consistently classified positive. Based on the standard deviation (1.2) estimated as the Root Mean-Squared Error (RMSE) from the linear regression, a sample with an average bacterial count of 50% is expected to show a bacterial count between 26% and 81% in 90% of an assumed large number of repeated measurements.

Supplementary Fig. 1. Visualization of individual bacterial count values versus average count calculated across 6 determinations (performed on square root-transformed data)

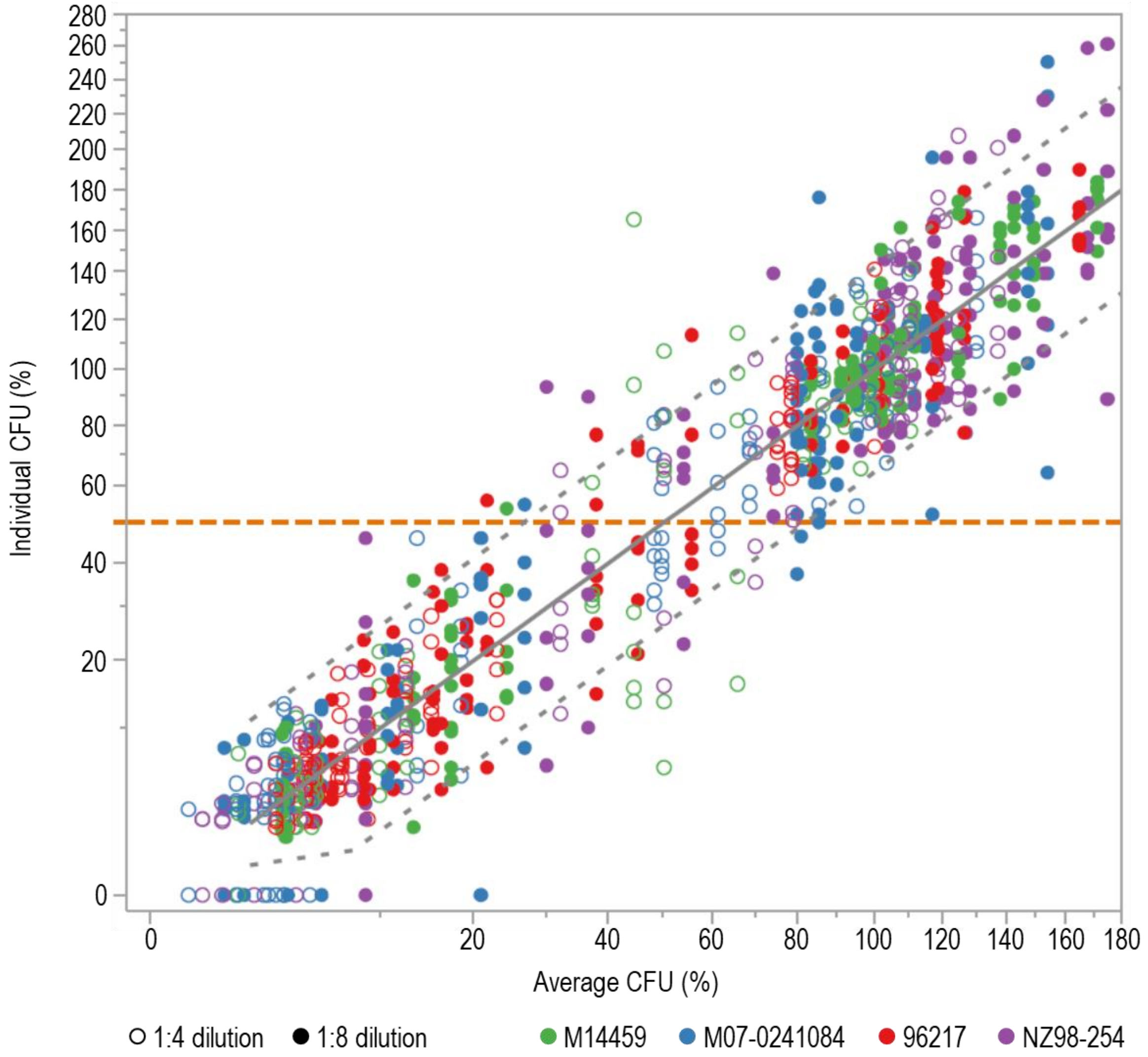

CFU, colony forming units.

Note: The solid and dashed lines show a linear regression with 2-sided 90% prediction bands, respectively.

Supplementary Table 1. Origin of serum samples used in the validation/qualification of enc-hSBA

Experiment(s)	Source	N
Qualification of the 110-strain panel	Study A (NCT01478347)	18
	Study B (NCT01911221)	12
Intermediate precision		
Pre-vaccination with 4CMenB	Study D	10
Post-vaccination with 4CMenB	Study A (NCT01478347)	9
	Marburg blood bank	1
Robustness		
Preservation of complement activity at different sample storage temperaturesa	Study E (NCT02140762)	20
Bacterial grow within the assay range	Study A (NCT01478347)	6
	Study D	3
	Marburg blood bank	2
Specificity	Study A (NCT01478347)	3
	Study B (NCT01911221)	1
	Marburg blood bank	2
Sensitivity		
Pre-vaccination with 4CMenB	Study D	18
Post-vaccination with 4CMenB	Study A (NCT01478347)	6
	Study C (NCT02305446)	5
MenACWY-positive samples	Study C (NCT02305446)	3
	Study D	10
	Marburg blood bank	9

enc-hSBA, serum bactericidal activity assay using endogenous human complement; N, number of serum samples; 4CMenB, 4-component meningococcal serogroup B vaccine; MenACWY, serogroup A, C, W, and Y meningococci.
